# Leveraging copper import by yersiniabactin siderophore system for targeted PET imaging of bacteria

**DOI:** 10.1172/jci.insight.144880

**Published:** 2021-05-24

**Authors:** Nabil A. Siddiqui, Hailey A. Houson, Nitin S. Kamble, Jose R. Blanco, Robert E. O’Donnell, Daniel J. Hassett, Suzanne E. Lapi, Nalinikanth Kotagiri

**Affiliations:** 1 Division of Pharmaceutical Sciences, James L. Winkle College of Pharmacy, University of Cincinnati, Cincinnati, Ohio, USA.; 2 Division of Advanced Medical Imaging Research, Department of Radiology and Chemistry, University of Alabama at Birmingham, Birmingham, Alabama, USA.; 3 Department of Internal Medicine, Heart, Lung and Vascular Institute, and; 4 Department of Molecular Genetics, Biochemistry and Microbiology, College of Medicine, University of Cincinnati, Cincinnati, Ohio, USA.

**Keywords:** Infectious disease, Bacterial infections, Diagnostic imaging

## Abstract

There is an emerging need for accurate and rapid identification of bacteria in the human body to achieve diverse biomedical objectives. Copper homeostasis is vital for the survival of bacterial species owing to the roles of the metal as a nutrient, respiratory enzyme cofactor, and a toxin. Here, we report the development of a copper-64–labeled bacterial metal chelator, yersiniabactin, to exploit a highly conserved metal acquisition pathway for noninvasive and selective imaging of bacteria. Compared with traditional techniques used to manufacture probes, our strategy simplifies the process considerably by combining the function of metal attachment and cell recognition to the same molecule. We demonstrate, for the first time to our knowledge, how a copper-64 PET probe can be used to identify specific bacterial populations, monitor antibiotic treatment outcomes, and track bacteria in diverse niches in vivo.

## Graphical abstract

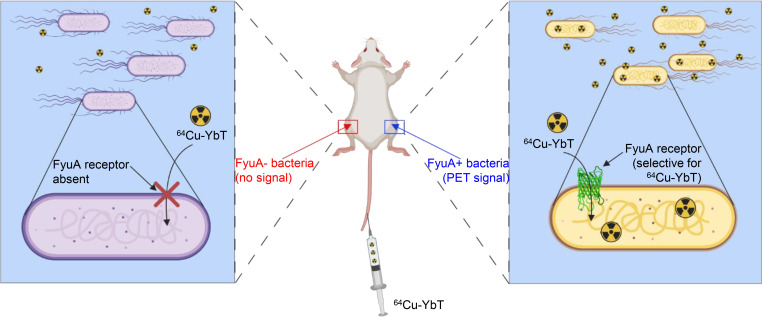

## Introduction

Noninvasive imaging of “live” bacteria in the body is in its infancy and a growing, critical, and unmet need, especially during problematic infections caused by multidrug-resistant bacteria. An effective approach should be able to accurately identify the correct pathogen and determine its distribution at the site of infection in order to minimize the indiscriminate use of both narrow- and broad-spectrum antibiotics. Numerous molecular imaging techniques, such as ultrasound, CT, MRI, single-photon emission CT, and PET, have been developed for preclinical and clinical research in the last 3 decades. However, current clinical probes, such as fluorine-18–labeled deoxyglucose (^18^F-FDG), cannot reliably distinguish bacteria from mammalian cells in vivo (1). Thus, a comprehensive understanding of bacterial physiology and genetics is required to develop probes for targeted imaging. Since bacteria are evolutionarily and phylogenetically distinct from mammalian cells, fundamental differences in metabolism and cellular structures can be leveraged to develop bacteria-specific imaging agents. Some of the recent approaches that have focused on targeting metabolic pathways and proteins unique to bacteria include ^11^C-para-aminobenzoic acid (2), involved exclusively in the bacterial folate pathway; ^18^F-maltohexaose (3), which specifically targets the maltodextrin transporter in bacteria; and ^18^F-fluorodeoxysorbitol, a synthetic analog of ^18^F-FDG that selectively localizes in Gram-negative *Enterobacteriaceae* (4). However, these radiotracers do not possess the ability to distinguish one genus or species from another. Aside from requiring high specificity and selectivity to the target bacterial population, labeling techniques for generating the reporter probes should be simple, rapid, and inexpensive to qualify as an ideal imaging agent. Current techniques employ a multistep process of labeling metabolites and ligands, which can lead to unnecessary delays and avoidable expenses, especially considering the short half-lives (*t_1/2_*) of some radionuclides.

**Figure 1:**
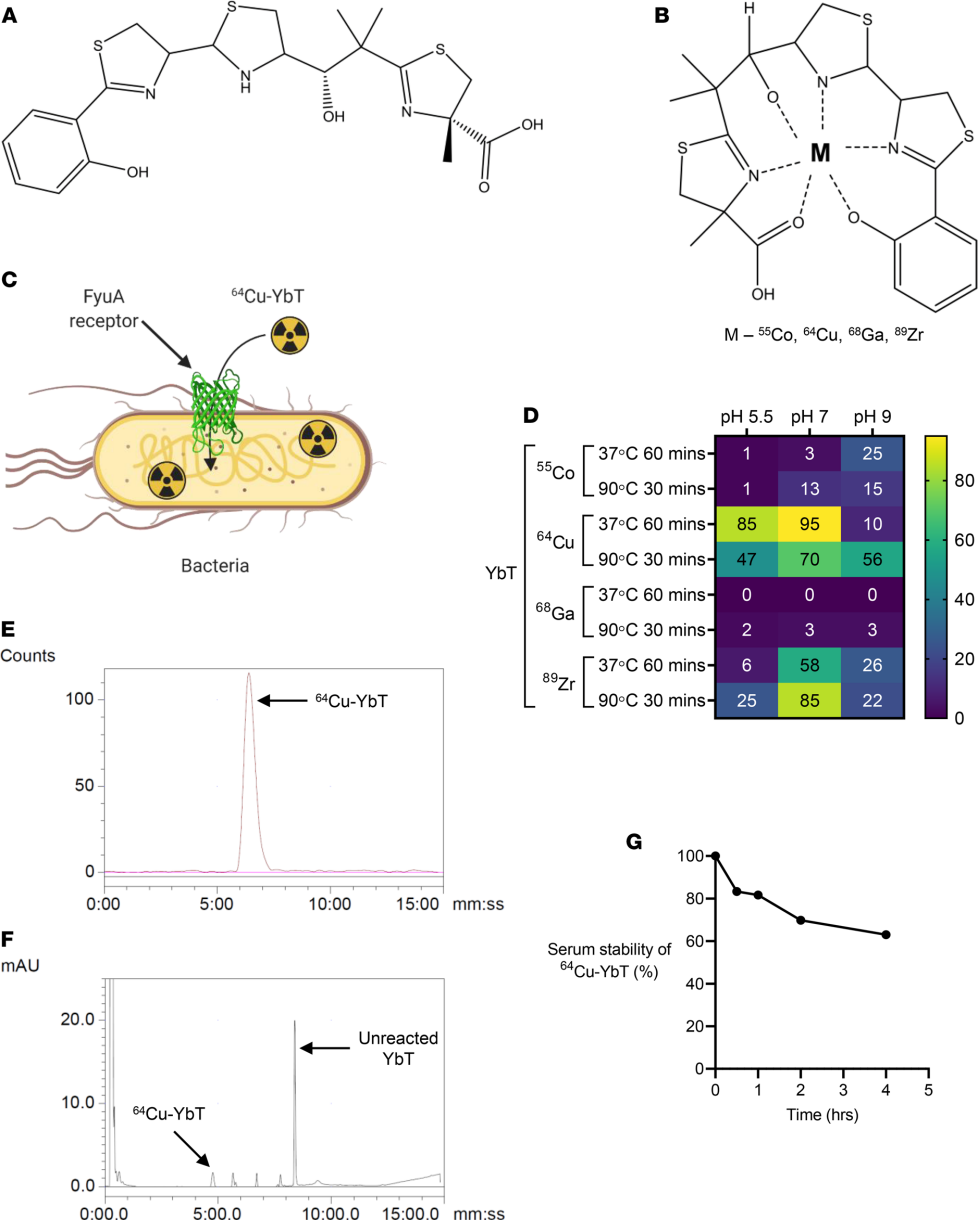
^64^Cu readily forms a complex with YbT with high affinity and stability. Chemical structures of (**A**) Yersiniabactin and (**B**) metal-YbT complex. (**C**) Schematic showing selective FyuA-mediated import of ^64^Cu-YbT inside bacteria. (**D**) iTLC characterization of YbT complexation (%) with ^55^Co, ^64^Cu, ^68^Ga, and ^89^Zr in various physicochemical conditions. (**E**) Radio and (**F**) UV HPLC chromatograms of ^64^Cu-YbT prepared at pH 7. (**G**) Serum stability of ^64^Cu-YbT at various time points after incubation.

Our study takes advantage of unique natural molecules that serve as both metal chelators and ligands, thus minimizing the labeling protocol to a simple 1-step process. Bacteria have developed sophisticated mechanisms to maintain metal homeostasis within a specified microenvironment. Entire pathways for metal acquisition — comprising (a) de novo synthesis of metal-binding peptide-like molecules known as siderophores and (b) dedicated membrane transporters that selectively bind the metal-siderophore complexes — have evolved to precisely regulate this process (5). The central role that these chelators play as chemical ligands in shuttling metals and the unique biology facilitating this transport into bacteria offers a platform that can be harnessed to develop highly versatile and specific contrast agents. In a recent study on the copper homeostasis of pathogenic *E*. *coli* UTI89, the siderophore, yersiniabactin (YbT) (Figure 1A), was shown to sequester and transport Cu(II) from the extracellular environment inside the bacteria (6). The Cu(II)-YbT/Fe(III)-YbT molar ratio in both human and mouse specimens was consistently greater than 1, suggesting that YbT binds host-derived copper at least as extensively as Fe(III) and demonstrating the physiological relevance of copper binding by YbT (6, 7). Metal-bound YbT (Figure 1B) is first selectively “recognized” by its cognate outer membrane protein receptor, ferric YbT uptake A (FyuA), before the complex is allowed cytoplasmic entry (Figure 1C). We hypothesized that FyuA-expressing bacteria can be targeted by copper-64–labeled (^64^Cu-labeled) YbT to facilitate imaging of bacteria with high specificity and sensitivity.

## Results

### ^64^Cu readily forms complexes with YbT with high affinity and stability.

To optimize the 1-step radiolabeling process, we labeled YbT with 4 different transition radiometals — ^55^Co, ^64^Cu, ^68^Ga, and ^89^Zr — in various buffered and temperature-controlled conditions. Primary characterization via instant thin-layer chromatography (iTLC) revealed highest complexation of YbT with ^64^Cu (Figure 1D). We elected to analyze ^64^Cu-YbT complexation in 0.1 M ammonium acetate buffer (pH 7) at 37°C further via high-performance liquid chromatography (HPLC). The presence of a single well-defined radio peak (Figure 1E) motivated us to choose this buffered media for probe preparation and all animal experiments. Since YbT was added in excess, a strong, sharp peak for the unreacted fraction was also recorded at around the same time by the UV detector at 220 nm (Figure 1F). For comparison and as a standard, we employed one of the most promising copper tracers used in preclinical and clinical studies, ^64^Cu-diacetyl-bis(N4-methylthiosemicarbazone) (^64^Cu-ATSM) (8). We noticed that ^64^Cu-ATSM exhibited slightly less efficient ^64^Cu binding compared with ^64^Cu-YbT (Supplemental Figure 1A; supplemental material available online with this article; https://doi.org/10.1172/jci.insight.144880DS1).

To examine the stability of ^64^Cu-YbT in vitro, we incubated the probe in mouse serum for 0, 0.5, 1, 2, and 4 hours. We used ethanol/ammonium acetate (50:50) as the extraction solvent and confirmed the presence of at least 60% of intact ^64^Cu-YbT 4 hours after the initial incubation (Figure 1G and Supplemental Figure 1B).

### ^64^Cu-YbT specifically identifies FyuA-expressing bacteria in vivo.

**Figure 2:**
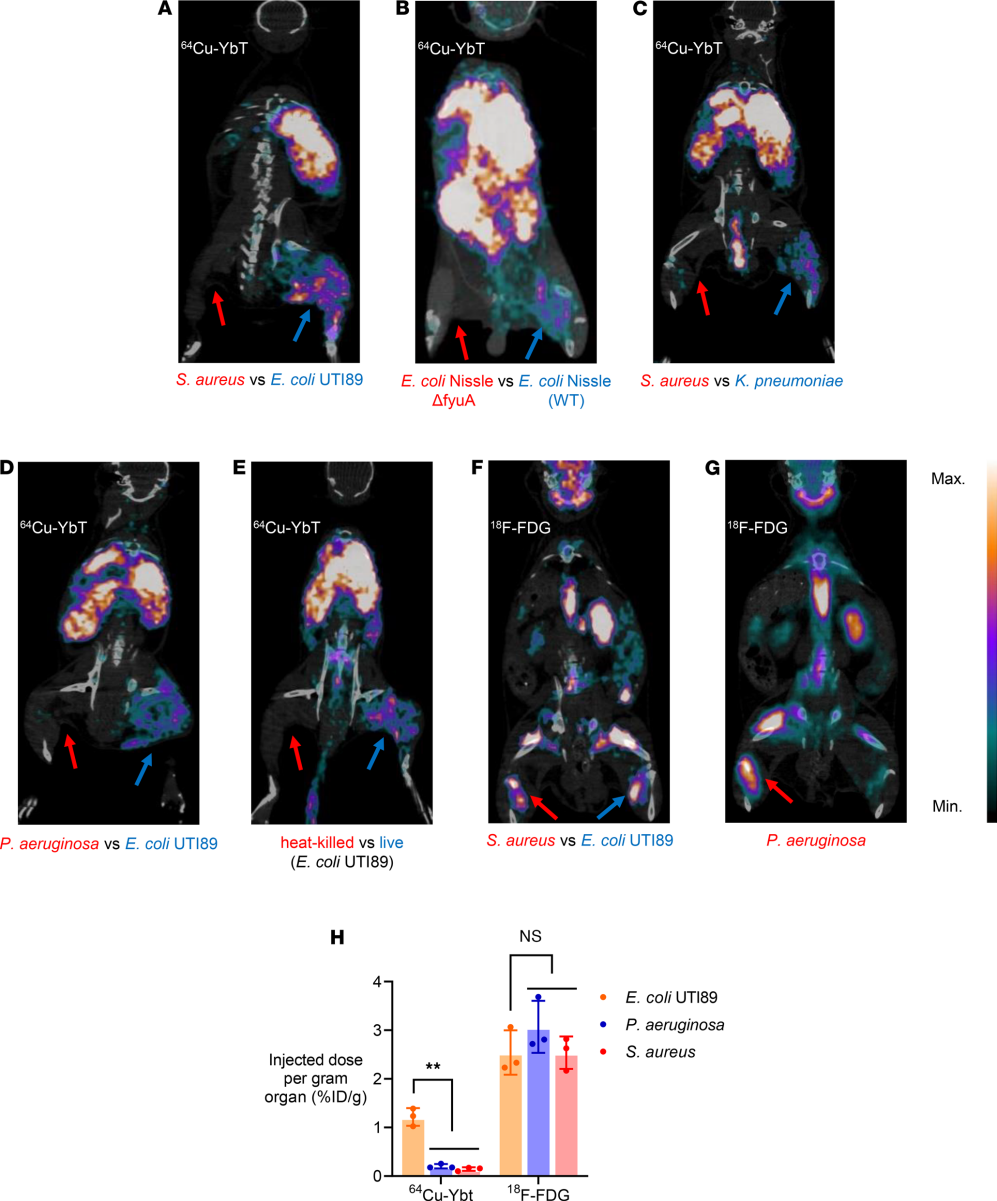
^64^Cu-YbT specifically identifies FyuA-expressing bacteria in vivo. PET/CT images of ^64^Cu-YbT 24 hours after administration in mice infected with (**A**) *S*. *aureus* and *E*. *coli* UTI89, (**B**) *E*. *coli* Nissle WT and *E*. *coli* Nissle FyuA-KO mutant (Δ*fyuA*), (**C**) *S*. *aureus* and *K*. *pneumoniae*, (**D**) *P*. *aeruginosa* and *E*. *coli* UTI89, and (**E**) live and heat-killed *E*. *coli* UTI89. PET/CT images of ^18^F-FDG 2 hours after administration in mice infected with (**F**) *S*. *aureus* and *E*. *coli* UTI89 and (**G**) *P*. *aeruginosa*. (**H**) Ex vivo biodistribution of ^64^Cu-YbT 24 hours and ^18^F-FDG 2 hours after administration in infected mice muscles. Note: arrows indicate sites of bacterial injection. Data are presented as mean ± SD (*n* = 3) and were analyzed by Greenhouse-Geisser’s 1-way ANOVA with Dunnett’s multiple-comparison test. **P < 0.01. NS, not significant.

Although FyuA-mediated import of ^64^Cu-YbT in *E*. *coli* UTI89 has previously been demonstrated in vitro (6), we sought to validate this phenomenon in vivo. To understand the general biodistribution of ^64^Cu-YbT, we administered ^64^Cu-YbT i.v. to naive mice. PET/CT imaging and ex vivo analysis revealed that ^64^Cu-YbT accumulated mostly in the liver and kidneys, with moderate uptake in the heart, lung, and the gastrointestinal tract and minimal uptake in brain, bones, and muscles (Supplemental Figure 2, A and B). We next administered ^64^Cu-YbT in mouse models with infectious myositis, generated by injecting bacteria into the hind limb muscles. Substantially higher PET signals (Figure 2, A–C) and activity (Supplemental Figure 2, C–E) were observed from muscles infected with FyuA-expressing *E*. *coli* UTI89 (6), *E*. *coli* Nissle WT, and *Klebsiella pneumoniae* (9, 10) compared with those infected with control bacteria that do not express a functional FyuA transporter protein. *Staphylococcus aureus* was used as control as it does not possess the YbT transport machinery. The other control bacteria was a KO mutant of *E*. *coli* Nissle (Δ*fyuA*), which we constructed by gene replacement of *fyuA* via the phage λ red recombinase technique.

After confirming the specificity of ^64^Cu-YbT toward FyuA-expressing bacteria, we sought to determine species selectivity of the probe, relative to *E*. *coli* UTI89. We selected *Pseudomonas aeruginosa*, a Gram-negative and multidrug-resistant nosocomial bacterium similar to *E*. *coli* UTI89 and noticed uptake of ^64^Cu-YbT more predominantly in muscles infected with *E*. *coli* UTI89 (Figure 2D). This is consistent with our previous observation in which bacteria, such as *P*. *aeruginosa* and *S*. *aureus*, do not uptake the probe due to absence of the FyuA transporter. Furthermore, to determine the correlation between bacterial viability (live vs. dead) and probe uptake, we administered ^64^Cu-YbT in mice infected with live and heat-killed *E*. *coli* UTI89 and observed signals only from muscles harboring live bacteria (Figure 2E). In addition, we performed comparative uptake studies involving a model of sterile inflammation using turpentine and noticed *E*. *coli* UTI89 specificity of ^64^Cu-YbT over turpentine-induced inflamed muscles (Supplemental Figure 2F).

### ^64^Cu-YbT exhibits higher specificity and comparable sensitivity with clinical PET probes.

**Figure 3:**
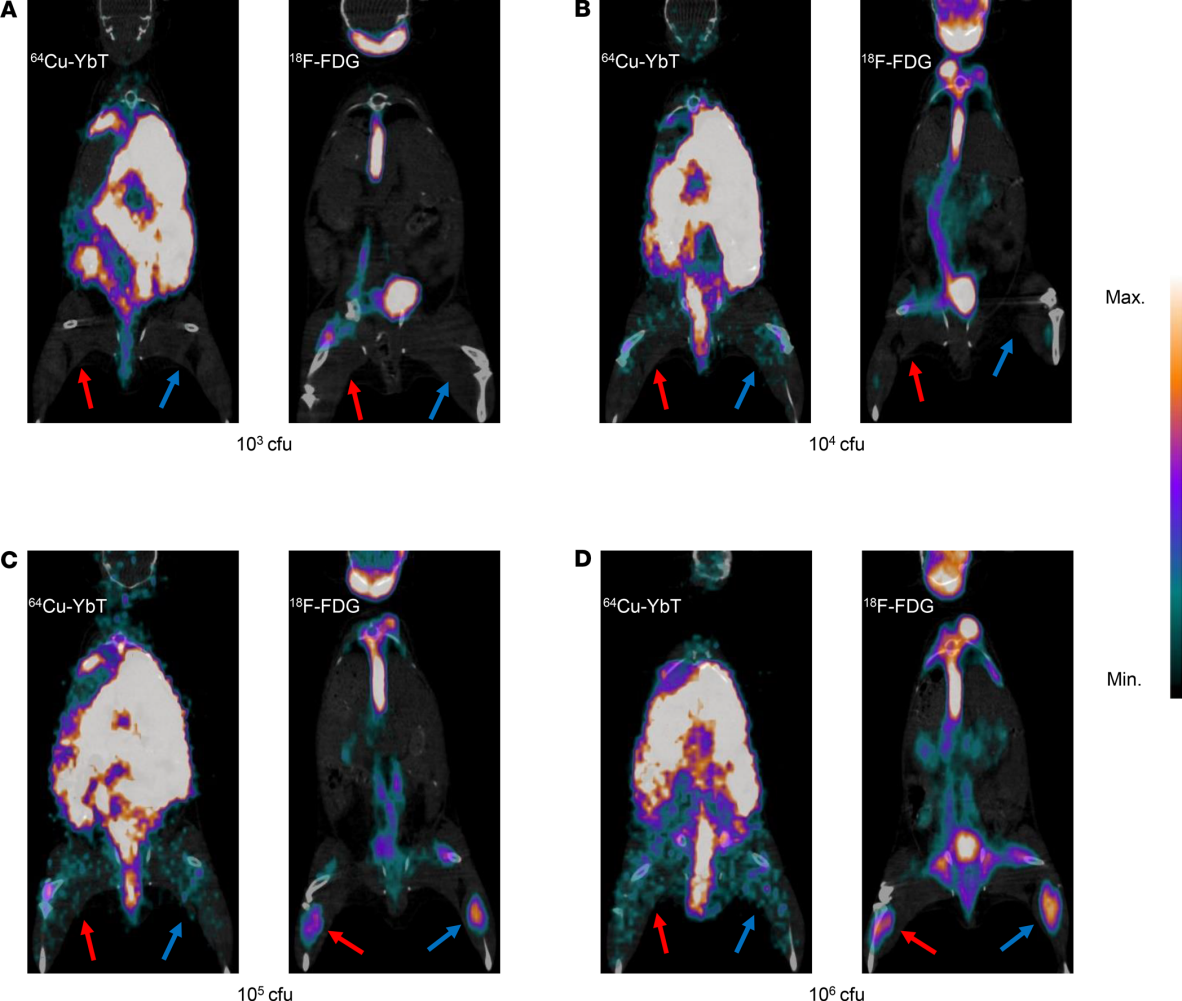
^64^Cu-YbT has comparable sensitivity to ^18^F-FDG. PET/CT images of ^64^Cu-YbT and ^18^F-FDG in mice infected with (**A**) 10^3^ cfu, (**B**) 10^4^ cfu, (**C**) 10^5^ cfu, and (**D**) 10^6^ cfu of bacteria 2 hours after administration of probes. Note: red arrows indicate *K*. *pneumoniae* and blue arrows indicate *E*. *coli* UTI89 injection sites.

Any new diagnostic probe must be evaluated against a reliable “gold standard,” so we compared the specificity of our probe with the clinically available ^18^F-FDG. We noticed that, unlike ^64^Cu-YbT, ^18^F-FDG accumulated uniformly in muscles harboring pathogenic bacteria, irrespective of their Gram specifications or species differences (Figure 2, F–H). Subsequently, we compared sensitivity of ^64^Cu-YbT to ^18^F-FDG by administering the probes in mice infected with 10^3^, 10^4^, 10^5^, and 10^6^ cfu of 2 different FyuA-expressing bacteria, *E*. *coli* UTI89 and *K*. *pneumoniae*. Figure 3 shows the rapid detection of as little as 10^4^ cfu of both *E*. *coli* UTI89 and *K*. *pneumoniae* by ^64^Cu-YbT within 2 hours, which is similar to the detection sensitivity of ^18^F-FDG. While neither ^64^Cu-YbT nor ^18^FDG was able to sufficiently detect a bacterial load ≤10^4^ cfu (Figure 3, A and B), their sensitivity was comparable in detecting bacterial loads >10^4^ cfu (Figure 3, C and D).

Although our in vitro data demonstrated reasonable stability of ^64^Cu-YbT in mouse serum (Figure 1G), we sought to explore the possibility of “free” ^64^Cu specifically localizing in muscles infected with FyuA-expressing bacteria by administering an exact dose of unchelated ^64^Cu in mice infected with *E*. *coli* UTI89 and *S*. *aureus*. We did not observe any significant uptake of “free” ^64^Cu in either of the muscles (Supplemental Figure 3A), nor was the whole-body biodistribution profile (Supplemental Figure 3B) any different from that of ^64^Cu-YbT. Next, we compared ^64^Cu-YbT with a non-YbT copper-chelated probe, ^64^Cu-ATSM. In spite of its widespread use in imaging hypoxia in numerous oncologic settings (11), ^64^Cu-ATSM is not known to follow any unique copper uptake pathway. Again, we did not observe any significant difference in uptake of the control probe (^64^Cu-ATSM) between the infected muscles (Supplemental Figure 3C), despite the similarity in its general biodistribution to that of ^64^Cu-YbT (Supplemental Figure 3D).

### ^64^Cu-YbT can be used to monitor antibiotic treatment efficacy.

**Figure 4:**
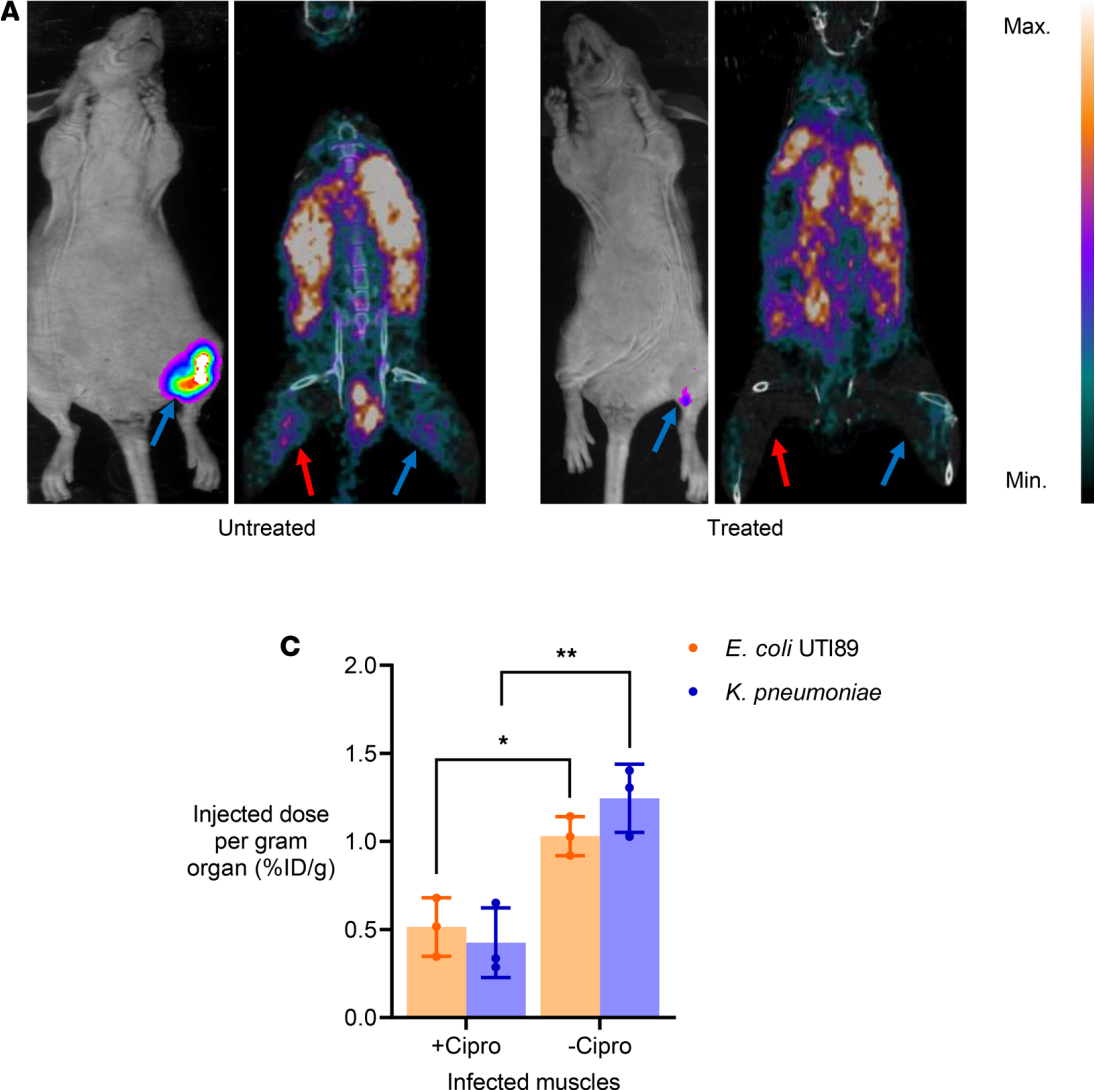
^64^Cu-YbT can be used to monitor antibiotic treatment efficacy. Bioluminescence followed by PET/CT images of (**A**) mice not treated with ciprofloxacin and (**B**) ciprofloxacin-treated mice infected with bacteria. (**C**) Ex vivo biodistribution of ^64^Cu-YbT 24 hours after administration. Note: red arrows indicate *K*. *pneumoniae* and blue arrows indicate *E*. *coli* UTI89 injection sites. Data are presented as mean ± SD (*n* = 3) and were analyzed by Welch’s *t* test. *P < 0.05, **P < 0.01.

Clearly, there are several advantages to having an imaging probe capable of performing “real-time” assessment of therapeutic success or failure of antibiotics for the management of seriously ill patients. To demonstrate whether ^64^Cu-YbT can potentially be used to monitor therapeutic outcomes, we injected ^64^Cu-YbT in mice infected with *E*. *coli* UTI89 and *K*. *pneumoniae* to image changes in signal intensity following administration of the broad-spectrum antibiotic, ciprofloxacin. We generated *E*. *coli* UTI89 clones with a luciferase reporter to track bacterial growth and burden using bioluminescence imaging (BLI). BLI was able to accurately indicate reduction in bacterial burden in response to antibiotic treatment (Figure 4, A and B). We used ^64^Cu-YbT to coregister PET signals with BLI and were able to show a decrease in PET signal corresponding to a proportional decrease in bacterial burden in mice that received 2 doses of ciprofloxacin (Figure 4B), compared with untreated mice (Figure 4A). When we harvested the thigh muscles from mice, gamma counter analysis revealed significantly lower activity from tissues of mice that received ciprofloxacin compared with the control mice (Figure 4C). These results demonstrate that accurate determination of antibiotic success can be achieved using ^64^Cu-YbT.

### ^64^Cu-YbT can specifically locate FyuA-expressing bacteria in diverse in vivo environments.

**Figure 5:**
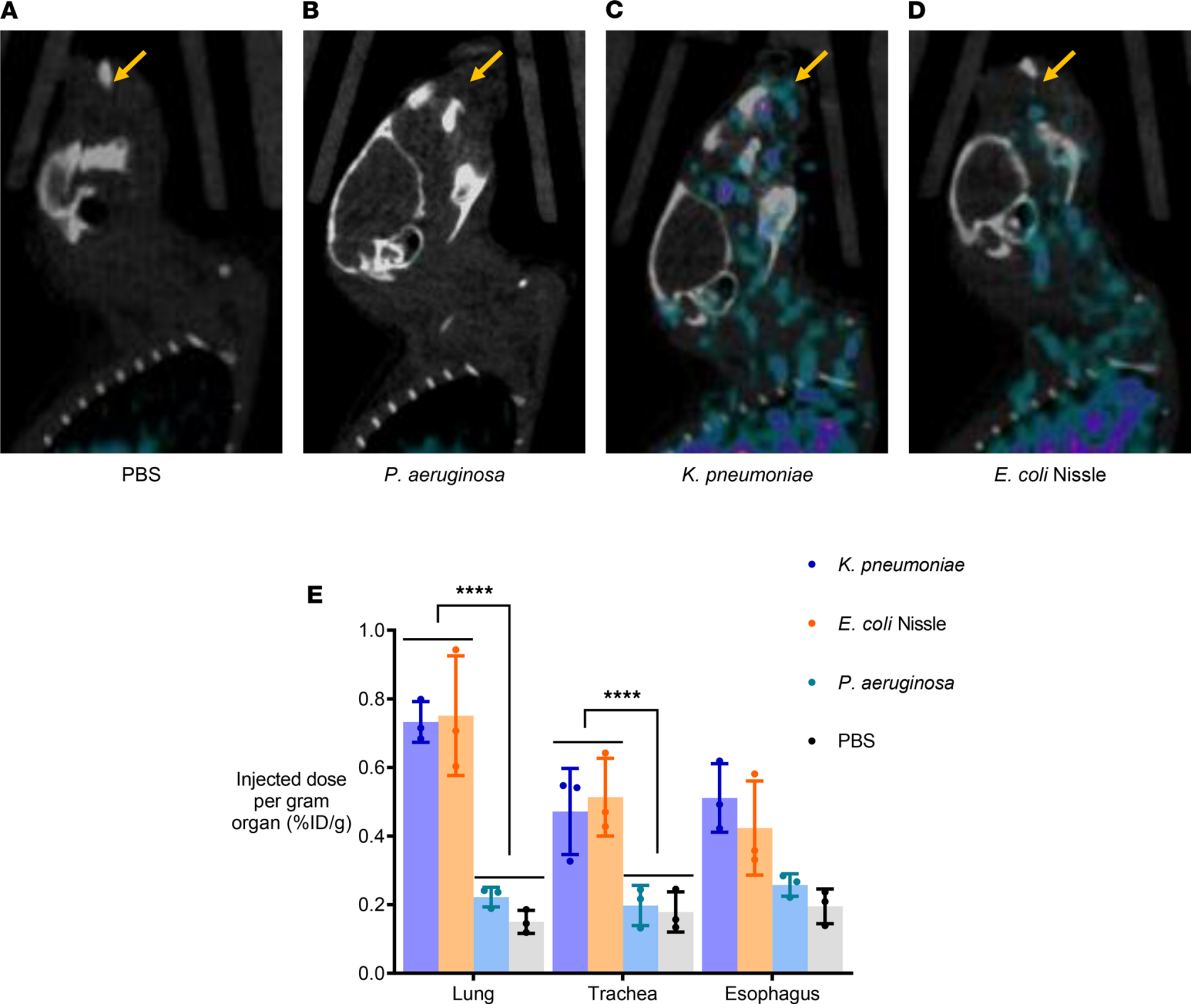
^64^Cu-YbT can specifically locate FyuA-expressing bacteria in diverse in vivo environments. PET/CT images of mice with intranasal administration of (**A**) PBS, (**B**) *P*. *aeruginosa*, (**C**) *K*. *pneumoniae*, and (**D**) *E*. *coli* Nissle. (**E**) Ex vivo biodistribution of ^64^Cu-YbT 24 hours after administration. Note: arrows indicate sites of bacterial administration. Data are presented as mean ± SD (*n* = 3) and were analyzed by 2-way ANOVA with Sidak’s multiple comparisons test. ****P < 0.0001.

Although we successfully demonstrated that ^64^Cu-YbT can specifically identify FyuA-expressing bacteria in myositis mouse models, we sought to investigate imaging of these bacteria in a clinically relevant model. We evaluated the efficacy of ^64^Cu-YbT in selectively identifying one particular bacteria, *K*. *pneumoniae*, in the lung. We developed our mouse models via intranasal administration of *E*. *coli* Nissle, *K*. *pneumoniae*, *P*. *aeruginosa*, and PBS (control). *P*. *aeruginosa*, which can also infect the lungs as an opportunistic pathogen, served as the primary negative control. *E*. *coli* Nissle served two purposes: (a) it served as a positive control and (b), owing to its nonpathogenic nature, it was used to dismiss any speculation of bacterial pathogenicity playing a role in selective ^64^Cu-YbT uptake. PBS was used as the vehicle to administer bacteria in the mice and, hence, served to establish a baseline for the study. After PET/CT imaging with ^64^Cu-YbT (Figure 5, A–D), supported by ex vivo biodistribution (Figure 5E) of harvested tissues, we observed significantly higher signals from lung and trachea of mice that received *K*. *pneumoniae* and *E*. *coli* Nissle compared with those that received *P*. *aeruginosa* and PBS. The mottled appearance of the PET signals from the nasal and thoracic cavity indicate and identify clusters of bacteria that dispersed from the point of origin to the trachea, lung, and esophagus. These results illustrate the ability of our probe to track and identify bacteria from the nasal confines to the pulmonary niche with a high degree of both spatial and temporal resolution.

## Discussion

In this study, we performed a comprehensive analysis of multiple radiometal-YbT complexes and successfully identified ^64^Cu-YbT as a highly stable PET probe that can selectively target bacteria with the cognate metal transport protein, FyuA. There is precedent for using siderophores as nuclear imaging contrast agents. ^68^Ga-labeled siderophores have been used in the past to image fungal infections (12–16). Coordination of ^89^Zr to desferrioxamine-bound (DFO-bound) vectors, such as antibodies, peptides, and nanoparticles, is a useful strategy for imaging tumor-specific receptors (17). More recently, ^68^Ga-DFO (18) and ^68^Ga-pyoverdine (19) probes have been used to image *S*. *aureus* and *P*. *aeruginosa* infections in animal models (20). In this study, we have validated that ^64^Cu-YbT, a metal-siderophore complex specific for certain bacterial populations, can be prepared with high purity and stability under physiological conditions within an hour. The probe can subsequently be used without further purification to selectively complete numerous clinically relevant bacteriological objectives in vivo. The 12.7-hour *t_1/2_* of ^64^Cu provides the flexibility to image at both shorter (2 hours) and longer (24 hours) time scales, allowing optimal clearance of the probe and obtaining images with high contrast (21). Logistically, the *t_1/2_* also allows the probe to be easily distributed for PET imaging studies at sites remote to the production facility with the loss of approximately 1 *t_1/2_*.

We have demonstrated the versatility of ^64^Cu-YbT to track pathogenic and probiotic bacteria in distinct in vivo settings; muscle and the lung. *E*. *coli* strains UTI89 and Nissle, *K*. *pneumoniae*, *P*. *aeruginosa*, and *S*. *aureus* were used for evaluating the probe in muscles. The rationale behind selecting the aforementioned bacteria was to demonstrate the highly selective uptake of ^64^Cu-YbT exclusively in bacteria expressing the FyuA transporter protein in vivo. In this setting, *E*. *coli* UTI89, *E*. *coli* Nissle, and *K*. *pneumoniae* express FyuA, while the rest represent negative controls. For clarity purposes, *E*. *coli* UTI89, *E*. *coli* Nissle, and *K*. *pneumoniae* are Gram-negative bacteria belonging to the *Enterobacteriaceae* family, of which *E*. *coli* UTI89 is a member of the uropathogenic subtype. In contrast, *P*. *aeruginosa* is a Gram-negative bacterium belonging to the *Pseudomonadaceae* family, while *S*. *aureus* is a Gram-positive bacterium belonging to the *Staphylococcaceae* family. The central premise of this study was that ^64^Cu-YbT has minimal cross-reactivity and has high selectivity across all major levels — family, genus, and serotype — of bacterial class stratification. This is in stark contrast to ^18^F-FDG, which is considered a gold standard in clinical infection imaging but is unable to distinguish different types of bacteria. Additionally, we ensured that ^64^Cu-YbT was taken up by an equivalent number (Table 1) of live FyuA-expressing bacteria and not nonspecifically in Δ*fyuA*, dead bacteria, and inflamed regions in the body. In a subsequent study to monitor ciprofloxacin treatment outcome using ^64^Cu-YbT, we demonstrated that infected muscles with live bacteria “lit up” due to the accumulation of the probe in mice that did not receive the antibiotic. However, no PET signals were observed in mice with identical infected muscles following antibiotic treatment. All this information would be vital in a healthcare setting, where a false positive signal by dead bacteria or inflammation caused by underlying medical conditions could potentially be avoided, preventing misdiagnosis and unnecessary antibiotic usage.

In the past, most PET probes have been developed to target *E*. *coli* and *S*. *aureus* (22). However, there have not been any significant effort toward the development of a selective imaging modality for the clinically important pathogen, *K*. *pneumoniae*. Therefore, we were interested in deciphering how rapidly and selectively ^64^Cu-YbT could provide a positive signal for FyuA-expressing *K*. *pneumoniae*. Hospitals, even in developed countries, run the risk of exposing patients to common pathogens, such as *K*. *pneumoniae*, *P*. *aeruginosa*, and methicillin-resistant *S*. *aureus*, that are known to cause many infections including ventilator-associated pneumonia (23). Particularly, carbapenemase-producing *K*. *pneumoniae* often prove fatal to patients when the bacteria migrate from the site of infection to the bloodstream, resulting in sepsis. Clinical isolates of *K*. *pneumoniae* have been shown to modulate immunometabolic pathways to enhance their survival and promote their dissemination in the host (24). Figures 3 and 5 illustrate how our probe can selectively and sensitively depict this phenomenon in vivo.

We have also demonstrated how ^64^Cu-YbT can potentially be used to track probiotic bacteria, such as *E*. *coli* Nissle, a popular bacterial template for various bioengineering applications (25, 26). Advances in genetic engineering have enabled the exploration of using nonpathogenic bacteria, particularly for cancer therapy, in the last 2 decades (27, 28). Selective tracking could allow scientists to better map its location, biodistribution, quantity, proliferation, turnover, and fate in the body and, hence, optimize the therapeutic potential of the engineered bacteria and accelerate the translation of bacterial therapies. While numerous optical imaging reporters have been used, bioluminescence and fluorescence have the inherent drawback of limited light penetration, unlike PET imaging, which is depth independent.

In conclusion, we have demonstrated the successful use of YbT as a “dual-role” compound — (a) a ^64^Cu chelator as well as (b) a targeting ligand for imaging and tracking of pathogenic and probiotic bacteria in vivo. The small probe size (~600 Da), when compared with conventional antibody or peptide-based PET probes, will potentially allow access through the lining of blood vessels to analyze extravascular structures with relative ease. One potential concern regarding the probe could be its specificity to a single bacterial receptor. For example, if the clinical objective is to locate any combination of *E*. *coli* UTI89, *E*. *coli* Nissle, or *K*. *pneumoniae*, it would be challenging to delineate the PET signals if both pathogenic and probiotic species are concentrated in or around the same region of the body. Nonetheless, we anticipate further research on the diverse array of natural siderophores as tailored contrast agents for imaging a wide range of pathogenic, probiotic, and engineered bacteria. The same probes can be used across diagnostic platforms for in vitro assays using highly sensitive liquid scintillation and gamma counters as well as in vivo via PET/single-photon emission CT/MRI imaging using appropriate radiometals. Combining siderophores with novel radioisotopes can provide unprecedented insights into unique radiochemistry techniques. For instance, complexation with manganese-52 could facilitate bacterial imaging with MRI or PET/MRI scanners to improve spatial resolution. Such innovations will reveal new and invaluable information on bacteria-host interactions in living systems, which in turn will help design advanced tools and future therapeutic strategies.

## Methods

### Chemicals.

All reagents were purchased from commercial sources as analytical grade and were used without further purification. YbT was purchased from EMC Microcollections GmbH. ^64^Cu was obtained from Mallinckrodt Institute of Radiology, Washington University School of Medicine, St. Louis, Missouri, USA. ^18^F-FDG was purchased from Cardinal Health.

### Bacterial strains and culture conditions.

*E*. *coli* Nissle (Mutaflor), strains *E*. *coli* UTI89, *P*. *aeruginosa* PAO1, *S*. *aureus* Mu50, and a clinical isolate of *K*. *pneumoniae* (provided in-house) were used in this study. All bacteria were grown in LB agar (BD Difco) plates or LB medium (BD Difco) at 37°C as appropriate. FyuA KO was performed in *E*. *coli* Nissle using the standard red recombinase method as previously described (Supplemental Figure 4A) (29). All primers (Table 2) were purchased from Integrated DNA Technologies Inc., and the plasmids (Table 3) were purchased from Addgene. High-fidelity and colony PCR were performed by using Phusion High Fidelity DNA polymerase (Thermo Scientific) and DreamTaq DNA polymerase (Thermo Scientific), respectively. Success of KO mutagenesis was confirmed by analyzing the colony PCR products of mutant and WT *E*. *coli* Nissle via gel electrophoresis (Supplemental Figure 4B). For BLI, *E*. *coli* UTI89 was transformed with pAKgfplux1 (Addgene) for constitutive expression of lux genes (30). The positive transformants were selected on Ampicillin plates before use.

### Radiometal-YbT complexation — primary characterization.

^55^Co, ^64^Cu, ^68^Ga, and ^89^Zr were produced at the University of Alabama. Complexation was performed by combining 50 μCi radiometal with 10 μg YbT in 50 μL of various buffered media. Samples were incubated at 37°C or 90°C for 1 hour or 30 minutes, respectively. Binding efficiency was determined via iTLC with 50 mM diethylenetriaminepentaacetic acid as the development buffer.

### ^64^Cu-YbT complexation — secondary characterization.

100 μCi (10 μL) of ^64^Cu (Mallinckrodt Institute of Radiology, Washington University School of Medicine, St. Louis, Missouri, USA) was mixed with 10 μg (10 μL) of YbT in 0.1 M ammonium acetate (pH 7) to bring the reaction volume to 100 μL. The mixture was vortexed for 10–15 seconds before incubating in a thermomixer with 800 rpm agitation at 37°C for 1 hour. Labeling efficiency and radiochemical purity were determined using radio-HPLC (Agilent 1260 Infinity II Quaternary System with a Flow-RAM radio-HPLC detector) on a C18 column (Agilent Poroshell 120 EC-C18 column, 3 × 50 mm, 4 μm). The mobile phase consisted of water (A) and acetonitrile (B) with a gradient of 5%–95 % B over 15 min using a flow rate of 1 mL/min. The retention time of ^64^Cu-YbT was 6.35 minutes, and a radiochemical yield of greater than 95% was achieved and used without further purification.

### Serum stability of ^64^Cu-YbT.

In vitro serum stability was conducted by incubating 1 μCi (10 μL) of freshly prepared ^64^Cu-YbT in 240 μL of mouse serum (Thermo Fisher) for 0, 0.5, 1, 2, and 4 hours. 250 μL ice-cold ethanolic ammonium acetate (50:50) was used to precipitate serum proteins at defined time points by vortexing for 10–15 seconds followed by centrifugation at 10,000*g* for 5 minutes. The supernatants were analyzed for ^64^Cu-YbT via HPLC using the method described above.

### Animal models.

Six- to eight-week-old female BALB/cJ mice (The Jackson Laboratory) (*n* = 3) were used for all experiments. Fresh cultures of bacteria were grown to an OD_600_ of 1.0 at 37°C in LB media while shaking at 200 rpm. Bacteria were pelleted at 6000*g* for 2 minutes and washed with PBS 3 times. For muscle infection, 10^3^ to 10^6^ cfu of bacteria in PBS were administered intramuscularly; for lung infection, 10^6^ cfu of bacteria were administered intranasally 24 hours before probe administration. To monitor antibiotic treatment efficacy, 2 doses (12 hours apart) of ciprofloxacin (10 mg/kg) were given to mice via oral gavage. This regimen was thought to provide a plasma peak level in the range that was above those required to achieve efficacy after an oral administration of 500 mg ciprofloxacin in humans (31). ^64^Cu-YbT was administered immediately after the second dose of ciprofloxacin.

### Imaging and ex vivo biodistribution studies.

100–150 μCi ^64^Cu-YbT or ^18^F-FDG in saline (0.9% sodium chloride) was injected i.v. in mice. Small-animal PET scan was performed 2 or 24 hours after injection on a μPET scanner (Siemens Inveon). Animals were placed in the supine position on the imaging gantry with continued warming for the duration of the scan. CT scan (80 kVp, 500 μA, at 120 projections) was acquired for anatomical reference overlay with PET image for a 15-minute acquisition with real-time reconstruction. PET images were acquired over an additional 15 minutes and spatial resolution in the entire field of view was determined by ordered subset expectation maximization in 2 dimensions. Histogramming and reconstruction were applied using Siemens Inveon software. Postprocessing was carried out with Inveon Research Workplace, and general analysis was used for contouring volume of interest (VOI). These VOI values were considered active infection volumes and used for further analyses. Bioluminescence images were acquired for 5 minutes using an IVIS Imaging System for quantification of radiance (total flux, photons per second [p/s]) of the bioluminescent signals from the regions of interest. After the imaging studies, the mice were euthanized via carbon dioxide inhalation and cervical dislocation. Organs and tissues of interest were removed and weighed. Residual radioactivity in the samples was measured with a gamma counter, and results are expressed as a percentage of injected dose per gram of organ (% ID/g). Muscles infected with each type of bacteria were homogenized and serially diluted in fresh LB media, before plating each dilution on an LB agar plate to determine the number of CFU that resulted from the infection.

### Statistics.

All statistical analyses were performed using GraphPad Prism 9 software. Two-tailed unpaired Student’s *t* test was performed to compare the means between 2 groups, and 1- and 2-way ANOVA were used for 3 or more groups. *P* < 0.05 was considered statistically significant.

### Study approval.

All animal experiments were performed under anesthesia (2% isoflurane), following protocols approved by the University of Cincinnati Biosafety, Radiation Safety, and Animal Care and Use Committees.

## Author contributions

NK conceived the project. NAS and NK designed the study. HAH performed primary characterization of radiometal-YbT complexation. NSK generated *E*. *coli* Nissle mutant. NAS performed secondary ^64^Cu-YbT characterization, stability, and in vivo experiments. JRB generated radionuclides. NAS and NK analyzed the data and wrote the manuscript with feedback from REO, DJH, and SEL.

## Supplementary Material

Supplemental data

## Figures and Tables

**Table 3:**
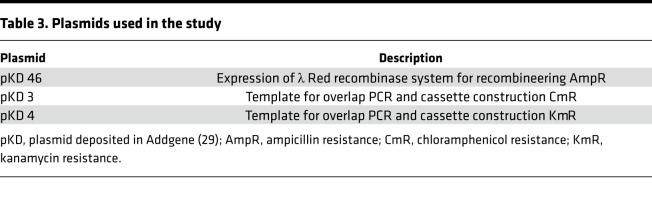
Plasmids used in the study

**Table 1:**
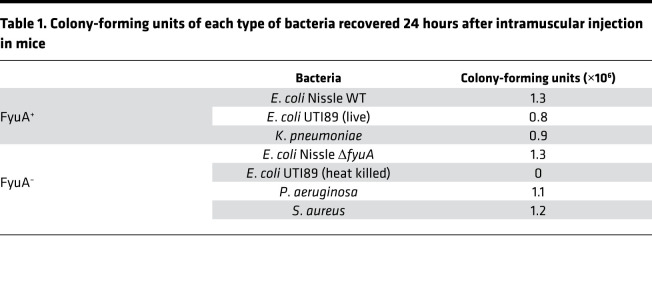
Colony-forming units of each type of bacteria recovered 24 hours after intramuscular injection in mice

**Table 2:**
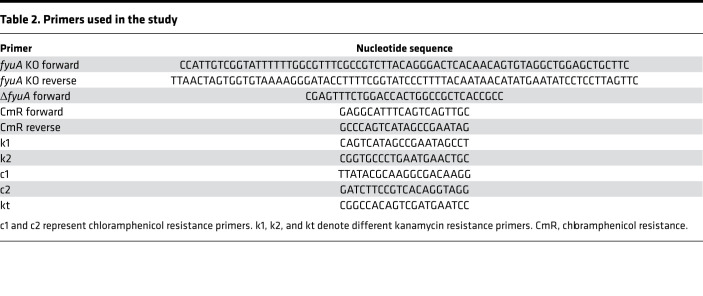
Primers used in the study

## Version 1 05/24/2021

Electronic publication

## Version 2 05/24/2021

The labels in Figure 3 were corrected
