# Supplementary material for: Leveraging copper import by yersiniabactin siderophore system for targeted PET imaging of bacteria
Source: JCI Insight. 2021 May 24;6(10):e144880. doi: 10.1172/jci.insight.144880 (PMC8262292; doi:10.1172/jci.insight.144880)
Supplement: Supplemental data [file jciinsight-6-144880-s298.pdf]

SUPPLEMENTAL DATA

Leveraging Copper Import by Yersiniabactin Siderophore System for Targeted PET Imaging of Bacteria

Nabil A. Siddiqui[1], Hailey A. Houson[2], Nitin S. Kamble[1], Jose R. Blanco[2], Robert E. O'Donnell[3], Daniel J. Hassett[4], Suzanne E. Lapi[2] & Nalinikanth Kotagiri[1]*

[1]Division of Pharmaceutical Sciences, James L. Winkle College of Pharmacy, University of Cincinnati, Cincinnati, Ohio, USA

[2]Division of Advanced Medical Imaging Research, Department of Radiology and Chemistry, University of Alabama at Birmingham, Birmingham, Alabama, USA

[3]Department of Internal Medicine, Heart, Lung and Vascular Institute, University of Cincinnati, Cincinnati, Ohio, USA

[4]Department of Molecular Genetics, Biochemistry and Microbiology, College of Medicine, University of Cincinnati, Cincinnati, Ohio, USA

*Corresponding author.

Supplemental Figure 1

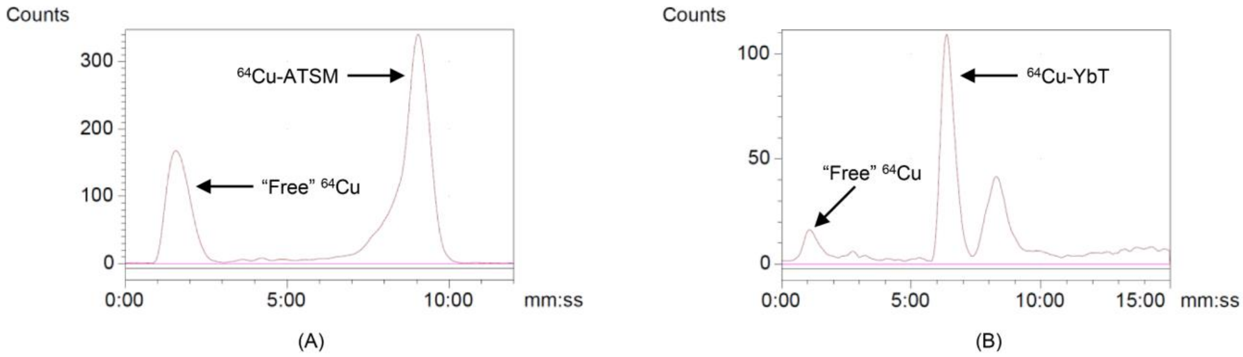

Supplemental Figure 1. HPLC analyses. Radio-chromatograms of (**A**) freshly prepared ^{64}Cu-ATSM and (**B**) ^{64}Cu-YbT in mouse serum 4 hrs after incubation.

Supplemental Figure 2

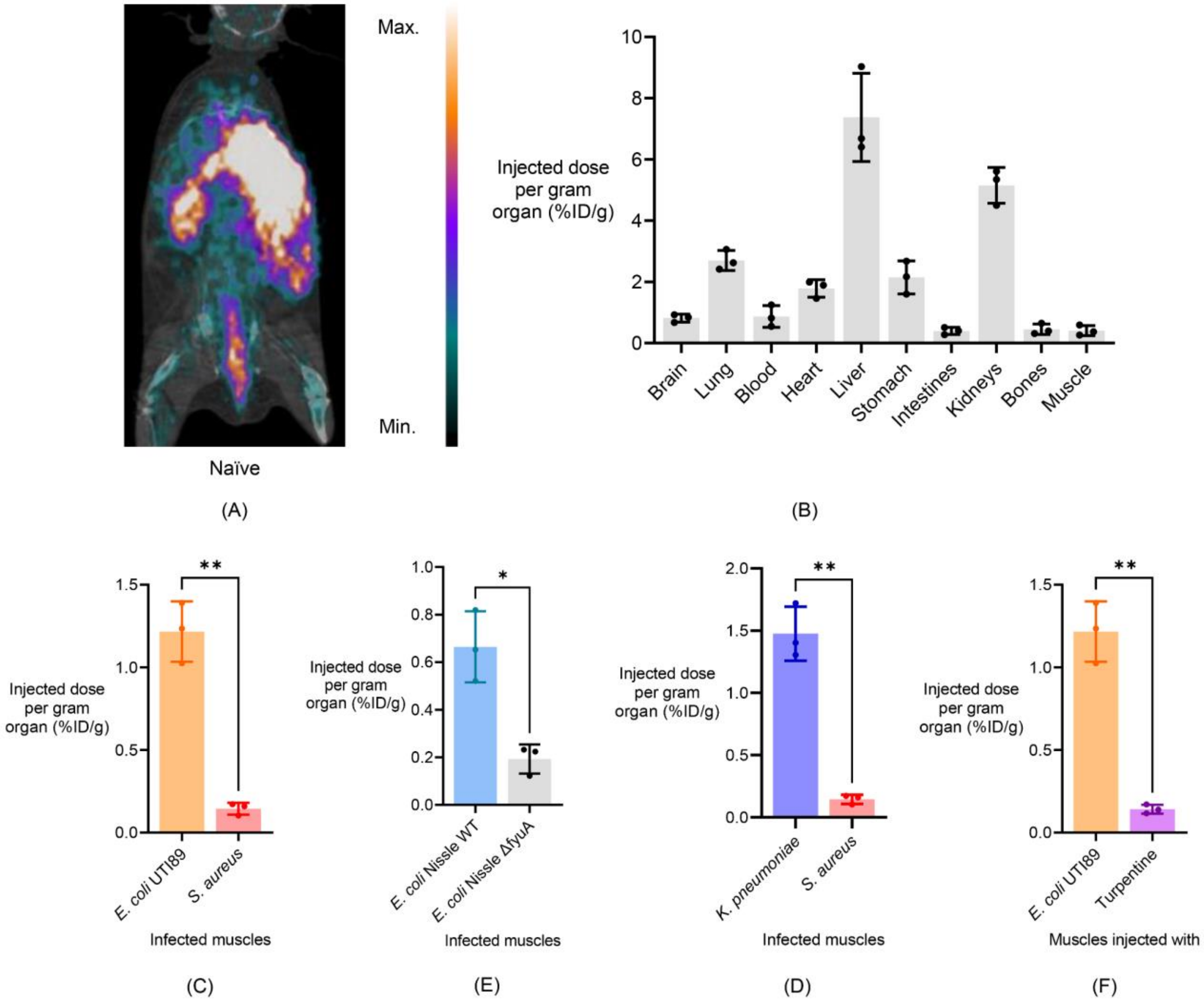

Supplemental Figure 2. *In vivo* profile of ^{64}Cu-YbT. (**A**) PET/CT image and (**B**) ex vivo biodistribution in major organs of naïve mice. ^{64}Cu-YbT accumulation (t = 24 hrs) in mice muscles injected with: (**C**) *E. coli* UTI89 and *S. aureus* (**D**) *E. coli* Nissle wild-type (WT) and *E. coli* Nissle FyuA knock-out mutant (Δ*fyuA*) (**E**) *K. pneumoniae* and *S. aureus* and (**F**) *E. coli* UTI89 and turpentine. Note: arrows indicate sites of bacterial injection. Data presented as mean ± s.d. (n = 3) analyzed by Welch's t-test; *P < 0.05, **P < 0.01.

Supplemental Figure 3

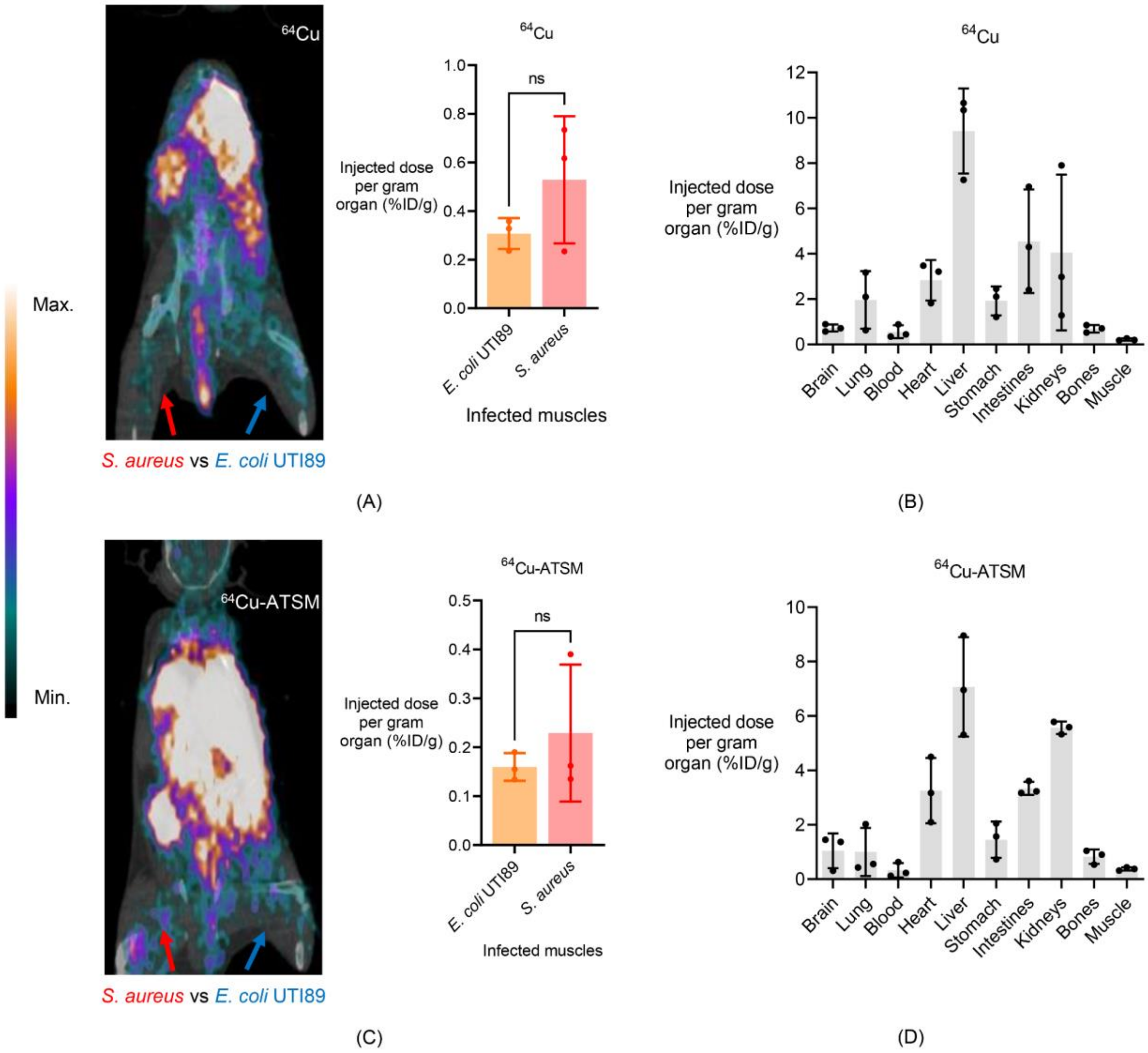

Supplemental Figure 3. Control ^{64}Cu-based probes lack bacterial specificity. PET/CT images with muscle uptakes and biodistribution in major organs of (**A** and **B**) Unchelated ^{64}Cu and (**C** and **D**) ^{64}Cu-ATSM 24 hrs post-administration of probes. Note: arrows indicate sites of bacterial injection. Data presented as mean ± s.d. (n = 3) analyzed by Welch's t-test; ns: not significant.

Supplemental Figure 4

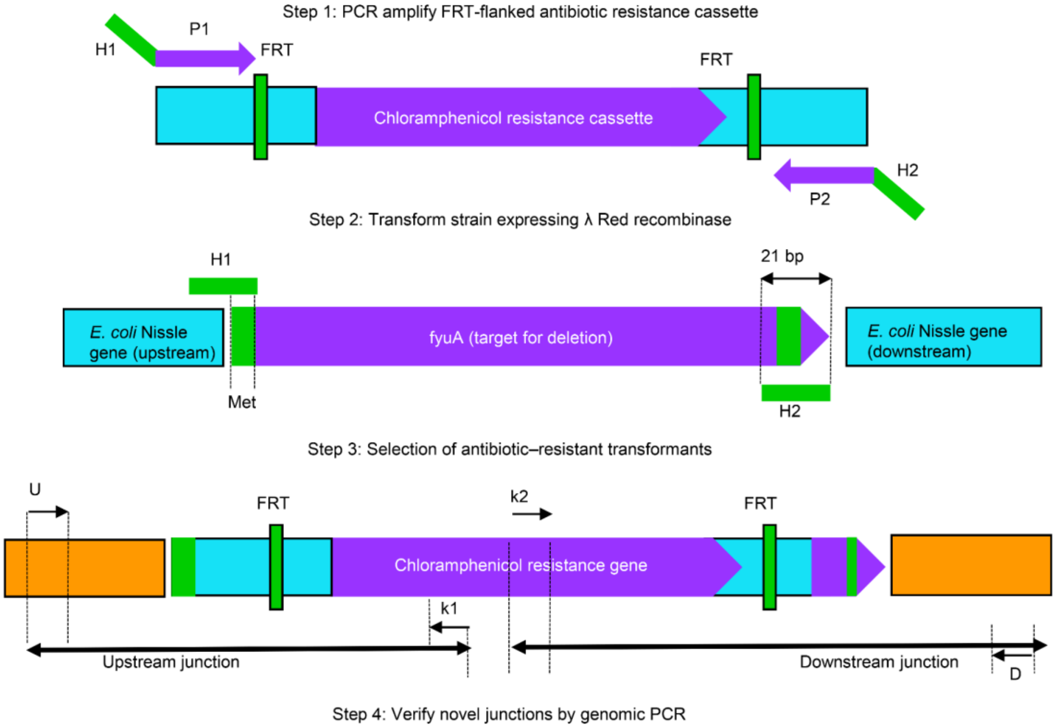

(A)

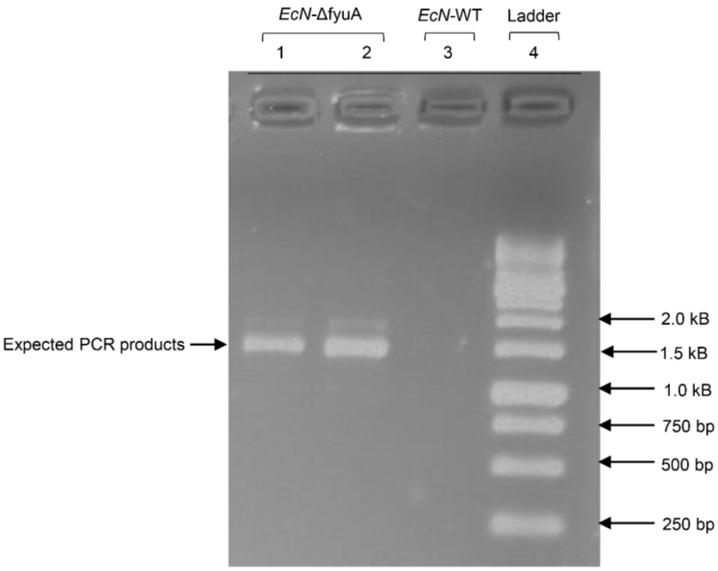

(B)

Supplemental Figure 4. (**A**) Schematic of knock-out mutant (KO) generation using λ Red Recombinase method. (**B**) Colony PCR products of *E. coli* Nissle FyuA KO mutant (*EcN*-Δ*fyuA*, lanes 1 and 2) and *E. coli* Nissle wild-type (*EcN*-WT, lane 3) were run on 1% agarose gel alongside a 1 kB DNA ladder.
